# Trajectories of Symptoms in Digital Interventions for Depression and Anxiety Using Routine Outcome Monitoring Data: Secondary Analysis Study

**DOI:** 10.2196/41815

**Published:** 2023-07-12

**Authors:** Diana Catalina Cumpanasoiu, Angel Enrique, Jorge E Palacios, Daniel Duffy, Scott McNamara, Derek Richards

**Affiliations:** 1 SilverCloud Science SilverCloud Health Dublin Ireland; 2 E-Mental Health Group School of Psychology Trinity College Dublin Dublin Ireland

**Keywords:** internet-delivered cognitive behavioral therapy, iCBT, depression, anxiety, trajectory of symptom change, routine outcome monitoring data

## Abstract

**Background:**

Research suggests there is heterogeneity in treatment response for internet-delivered cognitive behavioral therapy (iCBT) users, but few studies have investigated the trajectory of individual symptom change across iCBT treatment. Large patient data sets using routine outcome measures allows the investigation of treatment effects over time as well as the relationship between outcomes and platform use. Understanding trajectories of symptom change, as well as associated characteristics, may prove important for tailoring interventions or identifying patients who may not benefit from the intervention.

**Objective:**

We aimed to identify latent trajectories of symptom change during the iCBT treatment course for depression and anxiety and to investigate the patients’ characteristics and platform use for each of these classes.

**Methods:**

This is a secondary analysis of data from a randomized controlled trial designed to examine the effectiveness of guided iCBT for anxiety and depression in the UK Improving Access to Psychological Therapies (IAPT) program. This study included patients from the intervention group (N=256) and followed a longitudinal retrospective design. As part of the IAPT’s routine outcome monitoring system, patients were prompted to complete the Patient Health Questionnaire-9 (PHQ-9) and Generalized Anxiety Disorder-7 (GAD-7) after each supporter review during the treatment period. Latent class growth analysis was used to identify the underlying trajectories of symptom change across the treatment period for both depression and anxiety. Differences in patient characteristics were then evaluated between these trajectory classes, and the presence of a time-varying relationship between platform use and trajectory classes was investigated.

**Results:**

Five-class models were identified as optimal for both PHQ-9 and GAD-7. Around two-thirds (PHQ-9: 155/221, 70.1%; GAD-7: 156/221, 70.6%) of the sample formed various trajectories of improvement classes that differed in baseline score, the pace of symptom change, and final clinical outcome score. The remaining patients were in 2 smaller groups: one that saw minimal to no gains and another with consistently high scores across the treatment journey. Baseline severity, medication status, and program assigned were significantly associated (*P*<.001) with different trajectories. Although we did not find a time-varying relationship between use and trajectory classes, we found an overall effect of time on platform use, suggesting that all participants used the intervention significantly more in the first 4 weeks (*P*<.001).

**Conclusions:**

Most patients benefit from treatment, and the various patterns of improvement have implications for how the iCBT intervention is delivered. Identifying predictors of nonresponse or early response might inform the level of support and monitoring required for different types of patients. Further work is necessary to explore the differences between these trajectories to understand what works best for whom and to identify early on those patients who are less likely to benefit from treatment.

## Introduction

### Background

Depressive and anxiety disorders are 2 of the most common mental health difficulties, with epidemiological studies across countries suggesting that they are highly prevalent, can persist throughout lifetime, and are seriously impairing [1-4]. Cognitive behavioral therapy (CBT) is an effective psychological treatment regularly used to treat depression and anxiety symptoms [5,6]. In recent years, CBT has been adapted to an internet-delivered format (internet-delivered CBT [iCBT]) and has overcome some barriers associated with accessing traditional face-to-face psychological treatments [7,8]. Several meta-analyses have demonstrated the effectiveness of these interventions in treating depression and anxiety [9-13].

A key value in digitally delivered treatments is the ability to collect data on patient characteristics, routine outcome measures, and their engagement with the intervention. Given the volume of data collected, there is a potential new opportunity to understand treatment effects at an individual level. More than ever before, we can understand the trajectory of individual symptom changes and further explore the relationship between the use of the intervention and clinical outcomes. Understanding treatment effects over time and the relationship between use and outcomes may prove important for developing tailored interventions for different [14] patients. It may also be helpful in identifying patients who may be at risk of not responding, which can support clinical decision-making [15]. These efforts may have the potential to enhance digitally delivered treatments.

Most empirical evidence on trajectories of symptom change comes from face-to-face psychotherapy studies, which have found various classes of symptom courses, from early responders to late or delayed responders and steady or moderate responders [16,17]. Few studies have explored the evolution of symptoms during iCBT interventions. Some of them have consistently found a large group of users who show improvement and another group of users who show no or low symptom improvement [18,19]. Other studies have also found that most treatment responders experience the most clinical gains during the first weeks [20-22] and even before the treatment initiated [22]. Several of these studies also investigated the effects of individual baseline characteristics (eg, age and sex) on class membership; however, only symptom load has been consistently associated with class membership [20,21]. Similarly, studies have examined intervention use metrics and their relationship with class membership, with inconsistent findings reported [18,20,22]. While no differences were found between classes in terms of overall use time [18,22], two studies found differences between classes in the number of assessments, modules, and sessions completed [20,22].

In terms of intervention use and its relationship with outcomes from iCBT [23], it has been proposed that higher use (ie, better adherence or completion rate) predicts better outcomes [24]. However, other studies investigating the relationship between use metrics and outcomes have reported mixed results [25,26]. To date, many studies have been limited by their collection of outcomes at fixed time points. Evaluating use patterns in relation to continuous outcome monitoring may provide insight into how the temporal aspect of use is linked to changes in symptoms. Several studies [26,27] suggest that most use occurs at earlier stages of the intervention and that patients who improved have higher exposure levels to the intervention, especially in the first half [26]. A recent randomized controlled trial (RCT) presented an opportunity to examine the relationship between engagement and outcomes at different time points [28]. The results suggested that there was an association between use (completion rate and the frequency of items completed, but not time spent) and outcomes at 3 months but not earlier.

### Objectives

Overall, the current literature on trajectories of symptom change in iCBT is at an early stage, and more research is needed to confirm whether the classes found in previous studies are also observed in diverse samples from different settings. Identifying individuals who benefit from iCBT and those at risk of not improving is key to offering tailored interventions that fit the needs of specific populations, which may ultimately lead to increased response rates. In addition, learning more about the stage of treatment at which change occurs and its association with intervention use will shed light on whether intervention use acts as a mechanism for change in these trajectories. On the basis of this, this study sought to use routine outcome monitoring (ROM) data gathered from a pragmatic RCT in a clinical service setting to (1) identify latent classes of responders and associated participant characteristics during an iCBT intervention for depression and anxiety treatment and (2) investigate the presence of a time-varying relationship between trajectories of change and intervention use.

## Methods

### Study Setting

This study is a secondary analysis of data collected [29] at the Berkshire Healthcare Foundation Trust, a provider within the National Health Service Improving Access to Psychological Therapies (IAPT) program. IAPT is a stepped-care model in which people with depression and anxiety are offered different intensities of treatment depending on their needs and symptom severity. At step 2, clients are recommended low-intensity CBT-based treatments, such as guided self-help, internet-delivered CBT, or group CBT, under the supervision of a psychological well-being practitioner (PWP). The PWPs are a specially trained cohort of psychology graduate students, with additional qualification in delivering low-intensity CBT.  This study was conducted at step 2 of IAPT, with patients being assigned to iCBT as their preferred treatment option.

### Design

The original RCT where these data were collected was designed to examine the effectiveness and cost-effectiveness of the SilverCloud programs for anxiety and depression [29]. The study used a parallel-group design, in which an intervention group was compared with a waitlist control group, and the results demonstrated the effectiveness of the intervention group compared to the waitlist control after treatment, and improvements were sustained over a 12-month period [29].

Between June 28, 2017, and April 30, 2018, a total of 464 participants were invited to the original RCT; however, this study followed a longitudinal, retrospective design and included only patients from the intervention group (N=256). It captured the clinical assessments and platform use that occurred during the first 12 weeks of intervention use. While the main RCT used outcome data collected at research time points, this study used ROM data. As part of their treatment journey in IAPT, clients were prompted to complete the Patient Health Questionnaire-9 (PHQ-9) and Generalized Anxiety Disorder-7 (GAD-7) when they received a review approximately every 2 weeks. Hence, for each participant, assessments were available at baseline and at weeks 2, 4, 6, 8, 10, and 12.

### Participants

The eligibility criteria for this analysis mirrored that of the main RCT; therefore, to be included in the main RCT, a participant had to be aged >18 years, to present with mild to moderate symptoms of depression or anxiety, and to consent to engage with iCBT. In addition, comorbidity with psychotic illness, current psychological treatment, previous organic mental health disorder diagnosis, substance misuse, and suicidal risk (suicide-related thoughts, ideation, or active plans) were used as exclusion criteria.

### Interventions

On the basis of their symptoms and needs, clients were offered one of the following SilverCloud programs: Space from depression; Space from anxiety (different programs for specific anxiety disorders—modules for phobia, social anxiety, or generalized anxiety disorders); and Space from depression and anxiety, with the possibility of customizing treatment. Each participant had a PWP monitoring their progress and providing asynchronous reviews through the SilverCloud platform. The supporters had access to clients’ engagement and activity use through a dashboard interface, and they were encouraged to use this information to provide supportive and positive reviews to their clients.

All programs use evidence-based CBT principles and are delivered on the web (via a PC, tablet, or mobile device) on a Web 2.0 platform using media-rich interactive content (see Figures S1 and S2 in Multimedia Appendix 1, eg, for screenshots from the programs and prior publications [30] for more detailed descriptions). Each SilverCloud program has up to 8 modules, and it is recommended to complete 1 module per week. Each module incorporates quizzes, videos, informational content, interactive activities, homework assignments, and summaries. These interventions follow the National Institute for Health and Care Excellence guidelines [31] and have been tested and proved effective [30]. Participants were also assigned a PWP to support their progress through the program. These supporters monitor participants’ activities through the platform, provide guidance, and tailor feedback based on patient needs. This feedback comes in the form of regular reviews of the participants’ progress. In addition, supporters use these reviews to offer suggestions and guidance on how best to navigate the content and modules in each program to best fit an individual’s needs.

### Assessments

The data collected included demographic variables (age, sex, ethnicity, and employment), clinical measures for depression (PHQ-9) and anxiety (GAD-7), and use metrics.

The PHQ-9 is a brief, self-reported measure of depression [32,33] containing 9 items on a Likert scale (from 0 to 3). The score ranges from 0 to 27, with a cutoff score of ≥10 indicating the presence of depression and higher scores reflecting more severe symptoms. This assessment is widely used in clinical and research settings, and its validity, reliability (89%), sensitivity (88%), and specificity (88%) have been confirmed [32]. The GAD-7 is a brief, self-reported measure of anxiety [34] containing 7 items on a Likert scale (from 0 to 3). The score ranges from 0 to 21, with a cutoff score of ≥8 indicating the presence of anxiety and higher scores representing more severe symptoms. Similar to the PHQ-9, this assessment is widely used, and its validity and good internal consistency have been confirmed [34].

In terms of use, several objective metrics were obtained from the SilverCloud platform. Table 1 presents a full list of all use metrics and what each measured. To assess use at different time points in the treatment journey, all metrics were computed for each 2-week period between the start of treatment and week 12.

**Table 1:** Description of all usage metrics examined.

Use metrics	Descriptions
Number of log-ins	Number of log-ins for each participant adherence
Time spent	Length of time spent using the platform adherence
Number of reviews	Number of reviews each participant received from their psychological well-being practitioner engagement
Number of activities	Number of activities logged (eg, every time the participant used a tool or logged a journal entry)
Percentage of the program viewed	Percentage of new content viewed in each 2-week period engagement

### Procedure

For the main RCT, participants were first screened and then invited to participate in the study, and the consented participants were assigned to active treatment or waitlist. Once assigned to the active treatment group, participants were offered 1 of 3 SilverCloud programs (Space from Depression, Space from Anxiety, and Space from Depression and Anxiety) based on their needs. As the participants worked through the programs, they were presented with assessments to evaluate their progress. ROM is used to trigger assessments of participants at various time intervals to provide regular measurements of their progress. These assessments correspond with predetermined research time intervals that allowed for a deeper understanding of everyone’s journey through the program. All actions taken by the participants within the platform, such as module progress, content viewed, and activities completed, were collected through SilverCloud backend data collection.

### Analysis Plan

First, differences between the included and excluded participants were established using descriptive statistics (mean and SD) and independent 2-tailed *t* tests. To answer the first research question and identify trajectories of change, latent class growth analysis (LCGA) was used to identify latent classes. LCGA is a type of growth mixture modeling that is used to identify latent classes with different trajectories of growth. Mixture modeling approaches such as LCGA have been used more broadly in recent years because they allow the identification of underlying clusters based on unobserved heterogeneity in the data [35]. Compared with other growth modeling approaches that describe all trajectories with a single growth estimate, LCGA allows the identification of latent classes that have different characteristics (eg, intercept and slope) and assumes that all individual trajectories in a class are homogeneous [36-38]. This is done by fixing the variance of the intercept and slope within a class to 0 and allowing them to vary only across classes [37,38]. LCGA models address missing data using maximum likelihood algorithms [36,37]. To determine the optimal number of classes, models with an increasing number of classes are estimated, and different fit indices are used to compare them. There are multiple considerations taken into account when choosing the optimal model, such as the model fit indices, theoretical framework, clinical interpretation, and other criteria such as the number of participants in each class [36]. After the model is chosen, the probability of each individual to belong to one of the classes is estimated using maximum posterior probabilities and thus each individual is assigned to one of the latent classes.

LCGA is commonly conducted using statistical software, such as MPlus and SAS; however, recent efforts have made it easier to conduct such analyses in open-source R software [39]. For this analysis, the *lcmm* package [40] in R was used following the steps in the tutorial provided by Wardenaar [38]. A single-class growth model with a fixed intercept and slope for the subjects was initially run to test whether a linear, quadratic, or cubic model would be more appropriate for capturing the overall observed pattern of the trajectory. The coefficients of the cubic and quadratic terms had a poor model fit; therefore, a linear LCGA model was fitted. Latent class models were then constructed by increasing the number of classes from 2 to 8 to identify the optimal number of classes. Once a model was selected, 1-way ANOVAs and chi-square tests were performed to evaluate differences between classes in individual characteristics (eg, age, sex, and baseline severity).

Before investigating the relationship between use and symptom change, descriptive methods were used to explore use data, and regressions were run to understand the predictive values of different patient characteristics (eg, age and baseline severity) on each use metric. Then, to understand how different trajectories relate to use, mixed (between-factors and within-factors) ANOVAs were conducted to examine the role of time and class (PHQ and GAD) membership in use metrics. For the number of log-ins, length of use, number of reviews, number of activities, and percentage of programs viewed, five 3×5 mixed ANOVAs were conducted, with time as within-factor (3 levels: use in the first 4 weeks, 4-8 weeks, and 8-12 weeks) and class as between-factor (5 levels: the 5 trajectories identified). A total of 10 ANOVAs were run, 5 using the depression trajectories and 5 using the anxiety trajectories. All regressions and mixed ANOVAs were run using the R platform.

### Data Processing

As these were ROM data, it was decided to select the assignments completed on the closest date to the time points of interest (baseline, 2 weeks, 4 weeks, 6 weeks, 8 weeks, 10 weeks, and 12 weeks). The following criteria were used to do this: (1) an interval of –6 days to +6 days from each of the time points of interest was considered, and if there were more assignments done in that period, the one on the closest date to the time point of interest was selected; (2) if in the –6 days to +6 days interval there were 2 assignments equally close to the time point (eg, 1 assignment done 4 days before the time point and 1 assignment done 4 days after the time point), the second one was selected (ie, the one after the time point); and (3) if no assignments were completed in that time interval, a check was done to see if any assignments were done on the seventh day before or after the time point. If neither of these conditions was met, no assignment was selected for that time point.

As ROM data were used for outcomes with the criteria explained earlier, there were a number of missing PHQ-9 and GAD-7 assessments at each time point (for more details regarding missing data, see Table S1 in Multimedia Appendix 1). On average, each individual had 2 missed measurements out of 7 possible. There were also no significant results (all *P*>.05) from the logistic regression evaluating the predictive power of age, sex, baseline PHQ score, baseline GAD score, presence of a long-term condition (LTC), psychiatric medication status, and employment status on having more (defined as 3+) or less (defined as 0-2) missing assessments.

LCGA uses the maximum likelihood algorithm to handle both participants with full and missing data [36]. Missing data patterns for outcomes were evaluated and based on the Little test. After studying the different patterns of missing data, there was no evidence to disconfirm that data were missing at random; thus, analyses proceeded under this assumption. After identifying the latent classes, the pattern of missing data in each class was evaluated, and similar proportions of missing assessments were found in each class (Table S2 in Multimedia Appendix 1). For use, there were no missing data. Box plots were constructed to identify outliers, and the Winsorization method was applied. As use data are expected to be skewed, Winsorization was chosen over other methods (eg, truncating) to preserve all the data points and limit the influence of the extreme outliers. Several values were identified as potential outliers for the number of log-ins, length of use, and number of activities. All values determined as extreme (ie, 3 SDs or more away from the mean) were Winsorized to reduce the impact of those data points without removing them.

### Ethics Approval

This trial was approved by the National Health Service England Research Ethics Committee (reference: 17/NW/0311). The trial was prospectively registered at Current Controlled Trials (ISRCTN91967124).

## Results

### Overview and Sample Characteristics

Of the 256 participants in the intervention arm, 23 were excluded because they did not have a start date, and another 12 were excluded because of having only 1 assessment (the baseline). The analyses were conducted on the remaining 221 individuals.

Participants were aged 18 to 74 (mean 33, SD 12.68) years, had an overall baseline PHQ-9 score of 13.82, and had an overall baseline GAD-7 score of 12.26. Table 2 presents further descriptive information on the participants’ demographic and clinical characteristics.

**Table 2:** Patient characteristics (n=221).

Characteristics		Values
Age (years), mean (SD)		32.9 (12.68)
Baseline Patient Health Questionnaire-9 score, mean (SD)		13.82 (5.36)
Baseline Generalized Anxiety Disorder-7 score, mean (SD)		12.26 (4.97)
**Sex, n (%)**		
	Male	63 (29)
	Female	158 (71)
**Religion, n (%)**		
	No religious group or secular	143 (65)
	Other	72 (33)
	N/A^a^	6 (2)
**Sexual orientation, n (%)**		
	Heterosexual	193 (87)
	Other	20 (9)
	N/A	8 (4)
**Employment, n (%)**		
	Employed full time	166 (75)
	Other	55 (25)
**Psychiatric medication, n (%)**		
	Prescribed and taking	87 (39)
	Other	134 (61)
**L** **ong-term condition** **, n (%)**		
	No	176 (80)
	Yes	41 (19)
	N/A	4 (1)
**Program assigned, n (%)**		
	Comorbid	104 (47)
	Depression	50 (23)
	Anxiety	67 (30)

^a^N/A: not available.

The *t* test and Chi-square test comparisons showed no significant differences (all *P*>.05) between the 35 excluded participants and those included in terms of baseline PHQ-9 and GAD-7 scores and demographics, such as age, sex, religion, sexual orientation, employment status, psychiatric medication status, LTCs, and type of program.

### Latent Class Growth Analysis

Latent class models were constructed with an increasing number of classes, from 2 to 8 (Tables S3 and S4). The goodness-of-fit was assessed using Bayesian information criterion for each model to determine the optimal number of classes. In addition, the interpretability of the identified trajectories as well as their clinical meaningfulness were considered when choosing a model. On the basis of Bayesian information criterion index and theoretical considerations, the models with 5 classes were chosen for both the PHQ-9 and GAD-7.

### PHQ-9 Classes Description

**Figure 1:**
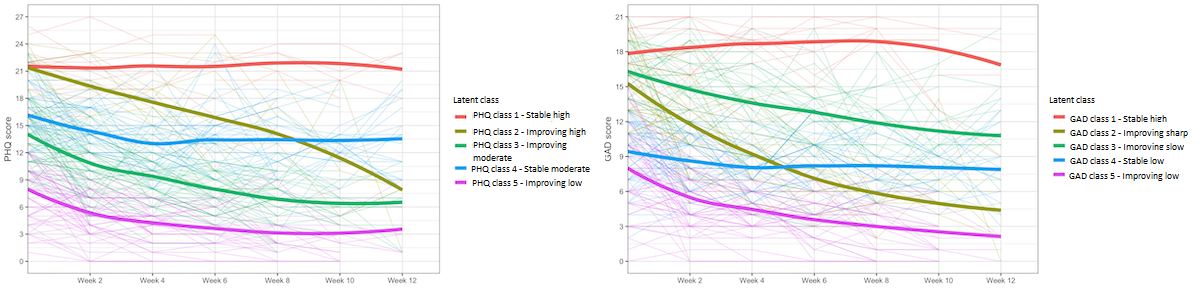
Trajectory classes for depression (left) and anxiety (right). GAD: Generalized Anxiety Disorder; PHQ: Patient Health Questionnaire.

A graphical representation of the 5 classes of depression trajectories can be found in Figure 1, where the individual patient trajectories and the mean trajectory for each class are shown. The characteristics of each class are summarized in Table 3. The 5 classes were as follows: “stable high” symptoms (10/221, 4.5%), “improving high” symptoms (17/221, 7.7%), “improving moderate” symptoms (77/221, 34.8%), “stable moderate” symptoms (56/221, 25.3%), and “improving low” symptoms (61/221, 27.6%). At baseline, the mean PHQ-9 scores for classes 1 (“stable high”) and 2 (“improving high”) were similar, but the patients in class 2 had a sharp decrease in symptoms across the treatment journey, whereas those in class 1 retained consistently high scores throughout treatment throughout the 12 weeks. Patients in class 3, “improving moderate,” showed a similar but slower decrease in symptoms to class 2 (“improving high”). Class 2 was the largest class, and the 77 individuals in it started with moderate PHQ-9 scores and consistently improved across the treatment journey, reaching a mean PHQ-9 score of 6.64 (SD 3.89) at 12 weeks. The patients in class 4, “Stable moderate,” started with moderate levels of depression and had a slight decrease in scores, remaining in the moderate range at the end of the intervention. Patients in class 5, “improving low,” started with subclinical levels at baseline and slowly but consistently decreased across the treatment journey.

**Table 3:** Patient Health Questionnaire-9 (PHQ-9) class characteristics.

		PHQ class 1 (“stable high”; n=10)	PHQ class 2 (“improving high”; n=17)	PHQ class 3 (“improving moderate”; n=77)	PHQ class 4 (“stable moderate”; n=56)	PHQ class 5 (“improving low”; n=61)
Participants (n=221), n (%)		10 (4.52)	17 (7.69)	77 (34.84)	56 (25.34)	61 (27.6)
Age (years), mean (SD)		30.1 (13.92)	34.47 (13.31)	31.31 (11.43)	32.38 (13.48)	35.41 (13.01)
Baseline PHQ-9 score, mean (SD)		21.6 (2.95)	21.41 (2.58)	14.12 (3.52)	16.05 (2.94)	8 (3.61)
Week 12 PHQ-9 score, mean (SD)		21.4 (2.07)	7.88 (3.64)	6.64 (3.89)	13.64 (3.55)	3.64 (2.10)
Baseline GAD-7^a^ score, mean (SD)		17.4 (3.47)	16.65 (3.00)	11.96 (5.03)	13.55 (4.32)	9.39 (4.14)
Week 12 GAD-7 score, mean (SD)		14.2 (5.31)	5.75 (3.11)	5.56 (3.80)	11.42 (4.46)	3.72 (3.58)
Sex (male), n (%)		1 (10)	6 (35)	25 (32)	16 (29)	15 (25)
Employment status (employed full time), n (%)		6 (60)	14 (82)	60 (78)	36 (64)	50 (82)
Psychiatric medication status (prescribed and taking), n (%)		7 (70)	12 (71)	29 (38)	25 (45)	14 (23)
Long-term condition (no), n (%)		8 (80)	12 (71)	61 (79)	45 (80)	50 (82)
**Program type, n (%)**						
	Comorbid	6 (60)	10 (59)	35 (45)	32 (57)	21 (34)
	Depression	1 (10)	6 (35)	21 (27)	14 (25)	8 (13)
	Anxiety	3 (30)	1 (6)	21 (27)	10 (18)	32 (52)

^a^GAD-7: Generalized Anxiety Disorder-7.

### GAD-7 Classes Description

A graphical representation of the 5 classes of anxiety trajectories can be found in Figure 1 (also Figure S3 in Multimedia Appendix 1), where the individual patient trajectories and the mean trajectory for each class are shown. The characteristics of each class are summarized in Table 4. The 5 classes were as follows: “stable high” symptoms (16/221, 7.2%), “improving sharp” symptoms (36/221, 16.3%), “improving slow” symptoms (54/221, 24.4%), “stable low” symptoms (49/221, 22.2%), and “improving low” symptoms (66/221, 29.9%). Class 1 (“stable high”) was the smallest, consisting of only 16 individuals whose trajectories of change were marked by consistently high scores from the baseline to 12 weeks. Patients in class 2 (“improving sharp”) and class 3 (“improving slow”) both started with severe anxiety, but the former had a much steeper decline in symptoms across the treatment journey. Patients in class 3, “improving slow,” started with severe levels of anxiety and had a slow and consistent decrease in scores. Class 4, “stable low,” consisted of participants who started and finished with mild levels with minimal changes in symptoms. Finally, patients in class 5, “improving low,” started with mild symptoms at baseline and slowly but consistently transitioned to minimal symptoms.

One-way ANOVAs and Tukey post hoc comparisons revealed significant differences between depression classes in baseline PHQ-9 (*F*_4,216_=89.21; *P*<.001) and baseline GAD-7 (*F*_4,216_=15.21; *P*<.001) scores. A similar difference was found between anxiety classes for both the baseline PHQ-9 (*F*_4,216_=23.14; *P*<.001) and GAD-7 (*F*_4,216_=82.59; *P*<.001) scores. Chi-square tests and Bonferroni-corrected pairwise comparisons also revealed significant differences between some of the depression classes regarding medication status (n=221, *χ*^2^_4_=18.5, *P*<.001) and the type of program (n=221, *χ*^2^_8_=25.7, *P*=.001). Depression class 5 (“improving low”) had a significantly lower percentage (14/61, 23%) of members with “prescribed and taking” medication compared with classes 2 (12/17, 70.6% “improving high”; *P*=.003) and 1 (7/10, 70.0% “stable high”; *P*=.003). Class 5 was also significantly different from classes 4 (“stable moderate”; *P*=.003) and 2 (“improving high”; *P*=.02) in terms of program type, with a much higher percentage (32/61, 52%) of members of class 5 being in the anxiety program compared with the other 2 classes where anxiety seemed to be the least common program (17.9% in the stable moderate class, and 5.9% in the improving high class). These results suggest that there are differences in the severity of depression and anxiety at baseline, with some of the identified PHQ and GAD classes starting with higher levels. Moreover, depression class 5 (“improving low”) seemed to distinguish itself from other depression classes by having more members in the anxiety program and a lower number of members taking medication. No other significant differences (all *P*>.05) were found between depression or anxiety classes in terms of age, sex, employment status, and the presence of LTCs.

**Table 4:** Generalized Anxiety Disorder-7 (GAD-7) class characteristics.

			GAD class 1 (“stable high”; n=16)		GAD class 2 (“improving sharp”; n=36)		GAD class 3 (“improving slow”; n=54)		GAD class 4 (“stable low”; n=49)		GAD class 5 (“improving low”; n=66)
Patients (n=221), n (%)			16 (7.24)		36 (16.29)		54 (24.43)		49 (22.17)		66 (29.86)
Age (years), mean (SD)			33.06 (13.16)		31.22 (10.78)		31.46 (12.48)		30.86 (11.57)		36.47 (13.74)
Baseline PHQ-9^a^ score, mean (SD)			19.69 (4.03)		15.08 (5.47)		16.65 (4.65)		12.43 (3.94)		10.42 (4.37)
Week 12 PHQ-9 score, mean (SD)			15.38 (5.34)		5.73 (2.99)		12.1 (4.60)		10.41 (5.92)		4.19 (3.31)
Baseline GAD-7 score, mean (SD)			17.88 (2.16)		15.28 (3.22)		16.31 (2.85)		9.41 (2.91)		8.06 (3.67)
Week 12 GAD-7 score, mean (SD)			17.13 (3.83)		4 (1.96)		10.97 (3.54)		8 (3.33)		2.08 (1.78)
Sex (male), n (%)			3 (19)		9 (25)		14 (26)		9 (18)		18 (27)
Employment status (employed full time), n (%)			12 (75)		28 (78)		39 (72)		37 (76)		50 (76)
Psychiatric medication status (prescribed and taking), n (%)			9 (56)		13 (36)		28 (52)		17 (35)		20 (30)
Long-term condition (no), n (%)			14 (88)		31 (86)		40 (74)		38 (78)		53 (80)
**Program type, n (%)**											
	Comorbid	11 (69)		15 (42)		28 (52)		27 (55)		23 (35)	
	Depression	1 (6)		5 (14)		11 (20)		13 (27)		20 (30)	
	Anxiety	4 (25)		16 (44)		15 (28)		9 (18)		23 (35)	

^a^PHQ-9: Patient Health Questionnaire-9.

### Platform Use

Overall, participants spent an average of 312 minutes on the platform, logged in approximately 18 times, viewed 57% of the total program, completed 150 activities, and received 4.26 reviews from their supporters (Table 5 provides descriptive information on platform use). It is noteworthy that there is large variability in all these use metrics, indicating considerable individual differences.

The results of the regressions indicate that age was a significant predictor of the number of log-ins (β=.13; *P*=.04), length of time spent on the internet (β=166.62; *P*=.03), and number of reviews (β=−.02; *P*=.009), whereas the presence of an LTC was a significant predictor of the number of activities (β=−43.5; *P*=.02), and the type of program was a significant predictor of the percentage of programs viewed (β=.07; *P*<.001). Overall, older people had a higher number of log-ins and spent more time on the platform but fewer reviews. The evaluation of box plots and descriptive summaries further showed that patients with an LTC had a higher number of activities compared with those without an LTC, and patients in the comorbid program had a smaller percentage of programs viewed compared with those in the depression or anxiety programs. No other baseline characteristics or demographic variables were found to be significant predictors of use (all *P*>.05).

**Table 5:** Platform use.

	Values, mean (SD)	Values, median (range)
Number of log-ins (Winsorized)	17.67 (11.13)	15 (1-70)
Length of use (Winsorized; minutes)	312.16 (228.83)	274.92 (2.47-1376.55)
Number of reviews	4.26 (1.63)	5 (0-8)
Number of activities (Winsorized)	150.48 (108.47)	126 (0-646)
Percentage of program viewed	0.57 (0.25)	0.58 (0.01-1)

### Relationship Between Outcomes and Use

Mixed ANOVAs were conducted to identify the effect of depressive symptom trajectory and time on use. There were no significant interactions of time by trajectory (all *P*>.05). For all 5 use metrics: the number of log-ins (*F*_2,432_=53.37; *P*<.001), length of use (*F*_2,432_=70.51; *P*<.001), number of reviews (*F*_2,432_=28.13; *P*<.001), number of activities (*F*_2,432_=56.20; *P*<.001), and percentage of program viewed (*F*_2,432_=86.34; *P*<.001), the ANOVAs revealed a significant effect of time. Paired *t* tests with *P*-adjusted Bonferroni post hoc analyses indicated that there were significant differences between all time points for all 5 use metrics (all *P*<.001), with most use occurring in the first 4 weeks, followed by weeks 4 to 8, and the least use occurring in the last 4 weeks (8-12). A detailed set of comparisons has been included as supplementary material for anyone who wants to delve deeper into the data (Tables S5 and S6 in Multimedia Appendix 1).

**Figure 2:**
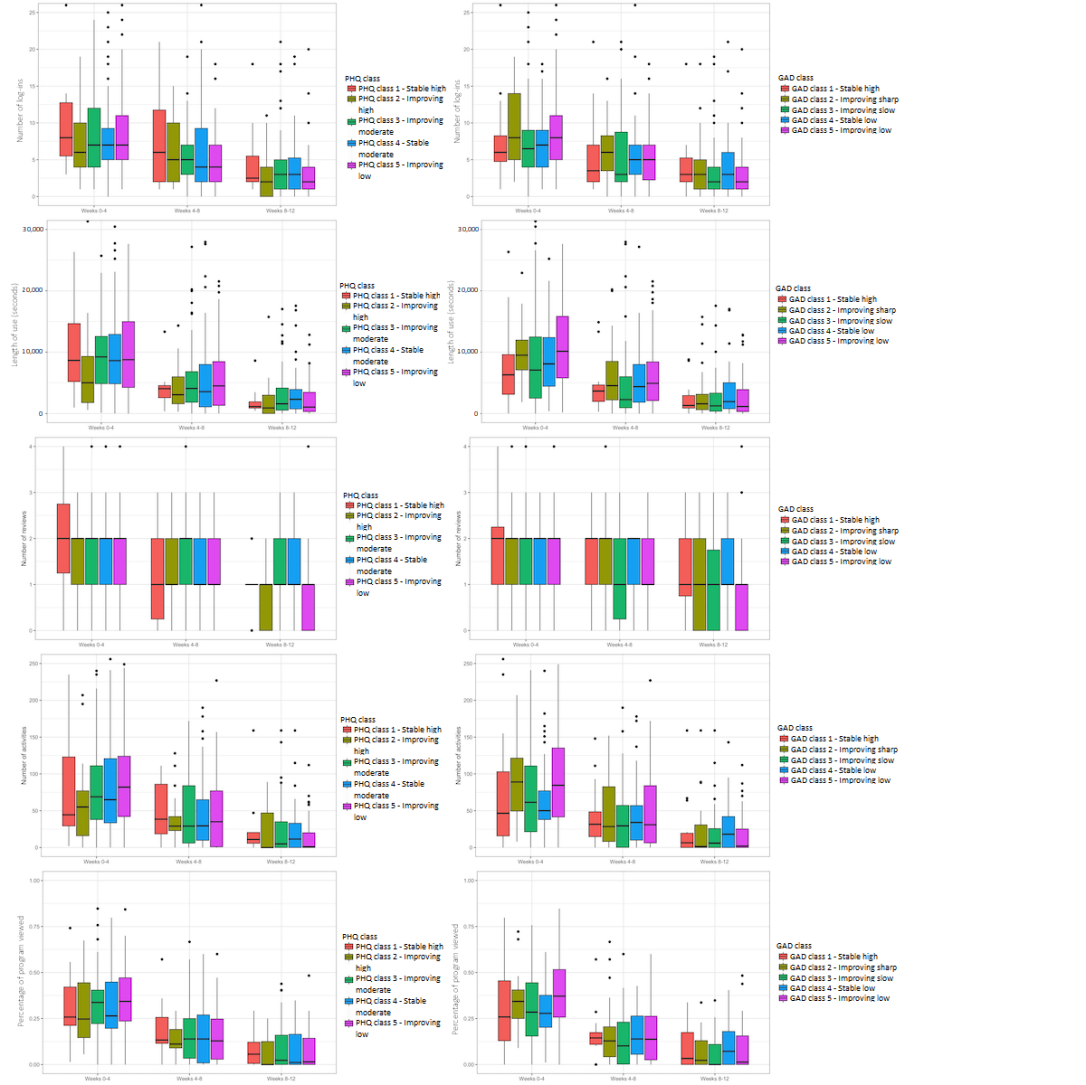
Differences in usage between trajectory classes at different time points. GAD: Generalized Anxiety Disorder; PHQ: Patient Health Questionnaire.

Mixed ANOVAs similar to the ones mentioned earlier were conducted to explore the effect of anxiety trajectory and time on usage. The Mauchly test demonstrated that the sphericity assumption had been violated, so Greenhouse-Geisser corrections were applied. There were significant interaction effects of time by trajectory for the number of log-ins (*F*_8,432_=1.99; *P*=.045) and number of activities (*F*_8,432_=1.98; *P*=.047); however, these effects were no longer significant after applying Greenhouse-Geisser corrections (*P*>.05). Interaction effects were not statistically significant for other use metrics (all *P*>.05). For the number of logins (*F*_2,432_=65.43; *P*<.001), length of use (*F*_2,432_=91.46; *P*<.001), number of reviews (*F*_2,432_=30.57; *P*<.001), number of activities (*F*_2,432_=85.62; *P*<.001), and percentage of programs viewed (*F*_2,432_=121.85; *P*<.001), the ANOVAs revealed a significant effect of time. . For all use metrics where a main effect of time was observed in the ANOVA, paired *t* tests with *P*-adjusted Bonferroni post hoc analyses indicated that there were significant differences between all time points (all *P*<.001), with most use occurring in the first 4 weeks, followed by 8 weeks, and the least at 12 weeks. Moreover, in the graphs for both depression and anxiety (Figure 2), it can be observed that there is substantial variability in use between the PHQ and GAD classes, where most of the variability between classes seems to occur in the early stages of treatment (first 4 weeks), and the differences between classes in the other 2 periods (weeks 4-8 and weeks 8-12) are less obvious. In addition, even after the Winsorization of the use metrics, the graphs allow us to see a high number of outliers.

## Discussion

### Principal Findings

This study aimed to identify different trajectories of symptom change across supported iCBT treatment for depression and anxiety using continuous outcomes data from patients treated in a mental health service setting. In addition, this study aimed to explore the relationship between the identified trajectories and use data. Overall, we found high heterogeneity in treatment response, with 5 latent classes emerging from data for both depression and anxiety. Across the 5 classes for depression and anxiety, we identified 3 improving classes and 2 classes that showed little to no change in symptoms. Across all classes, we also found an effect of time, with most use occurring in the first 4 weeks.

Understanding the different types of responses to an intervention as well as factors that may influence those responses could help improve the quality and delivery of iCBT interventions. This study found that approximately 70% of the sample improved across treatments; however, these individuals experienced different trajectories of change based on 3 characteristics: their baseline score, the pace of the improvement, and their posttreatment symptom scores. Some of the improver classes identified in this study are consistent with other iCBT studies that found individuals who started with moderate or moderate-severe scores and progressed toward recovery at a steady, moderate pace [19-22] and individuals who started with milder baseline symptoms and improved at a slower pace [20,21]. However, in general, our results showed steady improvements across improver classes instead of the early improvement classes observed in iCBT research [22]. These differences could be partially attributed to the nature of the ROM assessments linked to supporter reviews, as opposed to fixed time points. Of importance, we also found a class with severe anxiety (anxiety class 3) who, despite showing steady improvements, were still within the clinical definition of anxiety after treatment. Patients showing these trajectories could benefit from extending treatment or even adding high-intensity clinical interventions, such as face-to-face therapy, to support continued improvement that could help them achieve recovery [41].

It is perhaps even more important to understand the class characteristics of the individuals that see smaller to no gains so that in future, we could identify them early on, monitor their trajectory of symptoms, and make any necessary treatment decisions earlier to maximize treatment benefits. In particular, the 2 classes of nonresponders that start in the moderate severity range and see limited improvements (depression class 4 and anxiety class 4) could be ripe candidates for monitoring more closely symptom change trajectories and perhaps identify symptom change thresholds, whereby, from week to week, if thresholds are not met for symptom change, it could result in treatment decisions being made. Although some prior iCBT studies [19,22] have identified a class of limited to no improvement akin to depression class 4 and anxiety class 4 mentioned earlier, the nonresponder severe class found here has not been reported in other studies, perhaps because not many studies have included baseline scores on the severe end. It is possible that individuals in this severe nonresponder class need more support or to be stepped up in their care or that the interventions are unsuitable because they are primarily developed for mild to moderate ranges of symptom presentation. Therefore, the early identification of patients with high symptoms at baseline and an unchanged trajectory of symptom change early in treatment may also support better clinical decision-making.

It is also worth mentioning that we did not find a deterioration class similar to others [22], which could be a result of the intervention used here, or because of differences in the sample and methods used. In our primary RCT, where the current sample data originated, among 8-week measure completers, 5.2% (10/194) of participants in the intervention arm deteriorated (ie, increases in PHQ-9 score ≥6 or GAD-7 score ≥4) [42], which is in line with a recent individual patient data meta-analysis [43] that suggested only 5.8% of individuals in the intervention groups showed deterioration. Therefore, individuals with deteriorating trajectories in the current sample could be too few to create a subgroup of their own and may be mixed across the nonimproving classes.

A better understanding of the attributes of the classes identified has implications for tailoring, intervention delivery, and the early identification of individuals who are not on an improving path. We found no significant associations between the classes and baseline sociodemographic variables (ie, age, sex, employment status, and LTC), which is consistent with some studies [18,21], although other studies found that female individuals were more likely to be in the high-severity class [20]. Overall, the small classes with high interpersonal variability may have made it more difficult to identify differences between the groups, but future work could investigate other moderators related to clinical variables instead of demographic variables.

The results did not show a time-varying relationship between use and the various trajectories of change, indicating that the effect of platform use did not result in immediate clinical gains. This is consistent with findings from a study by Zeng et al [28], who only found this association by the end of treatment and therefore calls into question the potential role of use as a mechanism of change in iCBT. Studies with large observational samples should be conducted to detect these effects because the association between use and outcomes is more consistently found in large samples with aggregated data [12,24], whereas small-scale studies lead to more inconsistent findings [25,26]. However, we did find a consistent effect of time on the overall use of the intervention across classes and outcomes, suggesting that all participants used the intervention significantly more in the first 4 weeks. This is an important finding, as it replicates work from our group from a previous RCT [26], and the finding is consistent with other published literature [26,27,44]. Attention could be given to help maximize patients’ use of the intervention early in treatment. This can be achieved through frontloading key content or by increasing the schedule of support and guidance in the first 4 weeks.

The predictors of use were also investigated to better understand overall platform use. Age was a significant predictor for some of the use metrics, with older clients logging in more frequently and for longer periods, which is consistent with previous findings [27,45,46]. The content of the intervention may be better suited for older clients or older clients may need or have more time to read the material [45,46]. Alternatively, there may be a need to tailor the content in terms of cognitive load, delivery mode, and time commitment across different age groups.

### Limitations

A limitation of this study is the observational nature of this substudy, with no manipulation of the variables related to use, which makes it impossible to establish causal relationships between use metrics and outcomes. Moreover, using routine continuous outcome data compared with regular time point assignments comes with challenges, such as many missing questionnaires or complex data processing required to retrieve the relevant assignments. The small size of some of the classes presents another limitation, and future studies may benefit from larger sample sizes that could allow the detection of smaller effects and could provide an opportunity to apply other methods (eg, growth mixture modeling) to allow for both between- and within-class variability. Another limitation was the lack of access to baseline clinical information, which could have been useful for investigating differences between classes. For instance, variables that have been previously linked to treatment response include previous episodes of depression and anxiety [47], previous treatment or medication [48], client expectations [49], and treatment credibility [48,50]. Moreover, some of these clinical variables could be comorbidities and have a significant effect on the clusters found here. Further work is necessary to investigate and understand the role of these other clinical characteristics on the classes identified here.

### Conclusions

This study identified 5 distinct classes of symptom trajectories for depression and anxiety over the course of iCBT treatment. The results showed that although iCBT works for the majority, the way improvement occurs varies, which may have implications for how iCBT is delivered. The absence of effects on the time-varying relationship between platform use and trajectories calls into question the role of use as a mechanism for change. Other contextual information and larger sample sizes may need to be presented to explore these effects better. Further work is necessary to better understand these patterns of change, as well as factors impacting them as insights gained, which may be useful in tailoring treatments for different patient groups and in identifying and monitoring patient groups to enable earlier and enhanced treatment decisions.

## Appendix

Multimedia Appendix 1 Supplementary tables and figures with additional analysis information.

## Abbreviations

CBT: cognitive behavioral therapy
GAD-7: Generalized Anxiety Disorder-7
IAPT: Improving Access to Psychological Therapies
iCBT: internet-delivered cognitive behavioral therapy
LCGA: latent class growth analysis
LTC: long-term condition
PHQ-9: Patient Health Questionnaire-9
PWP: psychological well-being practitioner
RCT: randomized controlled trial
ROM: routine outcome monitoring
