# Supplementary material for: Trajectories of Symptoms in Digital Interventions for Depression and Anxiety Using Routine Outcome Monitoring Data: Secondary Analysis Study
Source: JMIR Mhealth Uhealth. 2023 Jul 12;11:e41815. doi: 10.2196/41815 (PMC10372559; doi:10.2196/41815)

**Multimedia Appendix 1. Supplementary tables and figures with additional analysis information**

**Table S1.** Missing data at each timepoint

|  | N | % |
| --- | --- | --- |
| Missing baseline PHQ | 0 | 0 |
| Missing week 2 PHQ | 53 | 0.24 |
| Missing week 4 PHQ | 48 | 0.22 |
| Missing week 6 PHQ | 74 | 0.33 |
| Missing week 8 PHQ | 69 | 0.31 |
| Missing week 10 PHQ | 123 | 0.56 |
| Missing week 12 PHQ | 114 | 0.52 |
| Missing baseline GAD | 0 | 0.00 |
| Missing week 2 GAD | 53 | 0.24 |
| Missing week 4 GAD | 48 | 0.22 |
| Missing week 6 GAD | 74 | 0.33 |
| Missing week 8 GAD | 69 | 0.31 |
| Missing week 10 GAD | 123 | 0.56 |
| Missing week 12 GAD | 115 | 0.52 |

**Table S2.** Missing data in each identified PHQ/GAD class

| PHQ class | N | Possible assessments | Missing PHQs | % missing PHQs | Missing GADs | % missing GADs | mean missing PHQ per person in class | sd missing PHQ per person in class | median missing PHQ per person in class | mean missing GAD per person in class | sd missing GAD per person in class | median missing GAD per person in class |
| --- | --- | --- | --- | --- | --- | --- | --- | --- | --- | --- | --- | --- |
| 1 | 10 | 70 | 16 | 0.23 | 16 | 0.23 | 1.6 | 1.07 | 1 | 1.6 | 1.07 | 1 |
| 2 | 17 | 119 | 40 | 0.34 | 40 | 0.34 | 2.35 | 1.46 | 2 | 2.35 | 1.46 | 2 |
| 3 | 77 | 539 | 172 | 0.32 | 173 | 0.32 | 2.23 | 1.39 | 2 | 2.25 | 1.41 | 2 |
| 4 | 56 | 392 | 114 | 0.29 | 114 | 0.29 | 2.04 | 1.29 | 2 | 2.04 | 1.29 | 2 |
| 5 | 61 | 427 | 139 | 0.33 | 139 | 0.33 | 2.28 | 1.32 | 2 | 2.28 | 1.32 | 2 |

| GAD class | N | Possible assessments | Missing PHQs | % missing PHQs | Missing GADs | % missing GADs | mean missing PHQ per person in class | sd missing PHQ per person in class | median missing PHQ per person in class | mean missing GAD per person in class | sd missing GAD per person in class | median missing GAD per person in class |
| --- | --- | --- | --- | --- | --- | --- | --- | --- | --- | --- | --- | --- |
| 1 | 16 | 112 | 32 | 0.29 | 32 | 0.29 | 2 | 1.10 | 2 | 2 | 1.10 | 2 |
| 2 | 36 | 252 | 80 | 0.32 | 80 | 0.32 | 2.22 | 1.40 | 2 | 2.22 | 1.40 | 2 |
| 3 | 54 | 378 | 129 | 0.34 | 129 | 0.34 | 2.39 | 1.43 | 2 | 2.39 | 1.43 | 2 |
| 4 | 49 | 343 | 93 | 0.27 | 93 | 0.27 | 1.9 | 1.31 | 2 | 1.9 | 1.31 | 2 |
| 5 | 66 | 462 | 147 | 0.32 | 148 | 0.32 | 2.23 | 1.29 | 2 | 2.24 | 1.31 | 2 |

**Table S3.** Patient Health Questionnaire-9 latent class models**.**

|  | Num of classes | Log likelihood | Entropy | Number of parameters | Akaike information criterion | Bayesian information criterion | Patients in class 1 (%) | Patients in class 2 (%) | Patients in class 3 (%) | Patients in class 4 (%) | Patients in class 5 (%) | Patients in class 6 (%) | Patients in class 7 (%) | Patients in class 8 (%) |
| --- | --- | --- | --- | --- | --- | --- | --- | --- | --- | --- | --- | --- | --- | --- |
| Lcga1 | 1 | −3379.24 | 1.00 | 3 | 6764.48 | 6774.67 | 100.0 | **—^a^** | **—** | **—** | **—** | **—** | **—** | **—** |
| Lcga2 | 2 | −3145.60 | 0.85 | 6 | 6303.20 | 6323.59 | 39.37 | 60.63 | **—** | **—** | **—** | **—** | **—** | **—** |
| Lcga3 | 3 | −3081.30 | 0.86 | 9 | 6180.61 | 6211.19 | 45.70 | 46.61 | 7.69 | **—** | **—** | **—** | **—** | **—** |
| Lcga4 | 4 | −3035.55 | 0.82 | 12 | 6095.11 | 6135.88 | 28.05 | 34.84 | 32.58 | 4.52 | **—** | **—** | **—** | **—** |
| Lcga5 | 5 | −3024.29 | 0.81 | 15 | 6078.59 | 6129.56 | 4.52 | 7.69 | 34.84 | 25.34 | 27.60 | **—** | **—** | **—** |
| Lcga | 6 | −3016.20 | 0.78 | 18 | 6068.40 | 6129.57 | 28.51 | 8.14 | 5.43 | 4.52 | 23.98 | 29.41 | **—** | **—** |
| Lcga7 | 7 | −3011.22 | 0.75 | 21 | 6064.65 | 6136.00 | 4.52 | 24.43 | 5.43 | 14.93 | 30.32 | 13.12 | 7.24 | **—** |
| Lcga8 | 8 | −3007.05 | 0.70 | 24 | 6062.10 | 6143.66 | 19.91 | 20.81 | 5.43 | 4.52 | 7.69 | 14.03 | 17.19 | 10.41 |

**‘—'** Not Applicable

**Table S4.** Generalized Anxiety Disorder-7 latent class models.

|  | Num of classes | Log likelihood | Entropy | Number of parameters | Akaike information criterion | Bayesian information criterion | Patients in class 1 (%) | Patients in class 2 (%) | Patients in class 3 (%) | Patients in class 4 (%) | Patients in class 5 (%) | Patients in class 6 (%) | Patients in class 7 (%) | Patients in class 8 (%) |
| --- | --- | --- | --- | --- | --- | --- | --- | --- | --- | --- | --- | --- | --- | --- |
| Lcga1.gad | 1 | −3254.81 | 1.00 | 3 | 6515.63 | 6525.82 | 100.00 | **—^a^** | **—** | **—** | **—** | **—** | **—** | **—** |
| Lcga2.gad | 2 | −3038.74 | 0.86 | 6 | 6089.48 | 6109.87 | 66.06 | 33.94 | **—** | **—** | **—** | **—** | **—** | **—** |
| Lcga3.gad | 3 | −2973.96 | 0.81 | 9 | 5965.92 | 5996.50 | 42.08 | 40.72 | 17.19 | **—** | **—** | **—** | **—** | **—** |
| Lcga4.gad | 4 | −2944.81 | 0.80 | 12 | 5913.62 | 5954.40 | 30.77 | 23.08 | 7.24 | 38.91 | **—** | **—** | **—** | **—** |
| Lcga5.gad | 5 | −2930.93 | 0.76 | 15 | 5891.87 | 5942.84 | 7.24 | 16.29 | 24.43 | 22.17 | 29.86 | **—** | **—** | **—** |
| Lcga6.gad | 6 | −2923.79 | 0.75 | 18 | 5883.58 | 5944.75 | 8.60 | 22.62 | 14.93 | 30.77 | 16.74 | 6.33 | **—** | **—** |
| Lcga7.gad | 7 | −2918.26 | 0.71 | 21 | 5878.52 | 5949.88 | 15.84 | 15.38 | 13.12 | 14.03 | 8.14 | 27.15 | 6.33 | **—** |
| Lcga8.gad | 8 | −2907.78 | 0.75 | 24 | 5863.56 | 5945.11 | 23.98 | 28.51 | 14.03 | 4.98 | 9.50 | 6.33 | 4.07 | 8.60 |

**‘—'** Not Applicable

**Table S5.** Differences in number of logins between different PHQ classes

| group 1 | group 2 | N1 | N2 | mean difference | CI | statistic | df | p | p adjusted | Cohen's D | CI |
| --- | --- | --- | --- | --- | --- | --- | --- | --- | --- | --- | --- |
| PHQ class 5 | PHQ class 4 | 61 | 56 | -1.22 | [-5.47, 3.04] | 0.57 | 113 | 0.27 | 1 | 0.11 | [-0.26, 0.49] |
| PHQ class 2 | PHQ class 4 | 17 | 56 | -0.99 | [-8.28, 6.29] | 0.28 | 24.6 | 0.78 | 1 | 0.08 | [-0.42, 0.69] |
| PHQ class 3 | PHQ class 4 | 77 | 56 | -0.96 | [-4.79, 2.87] | 0.49 | 104 | 0.62 | 1 | 0.09 | [-0.26, 0.44] |
| PHQ class 1 | PHQ class 4 | 10 | 56 | 4.17 | [-5.97, 14.3] | -0.9 | 11.5 | 0.39 | 1 | -0.32 | [-1.06, 0.4] |
| PHQ class 2 | PHQ class 5 | 17 | 61 | 0.22 | [-6.96, 7.4] | -0.06 | 23.2 | 0.95 | 1 | -0.02 | [-0.56, 0.56] |
| PHQ class 3 | PHQ class 5 | 77 | 61 | 0.26 | [-3.35, 3.86] | -0.14 | 119 | 0.88 | 1 | -0.02 | [-0.39, 0.3] |
| PHQ class 1 | PHQ class 5 | 10 | 61 | 5.38 | [-4.69, 15.46] | -1.17 | 11.1 | 0.27 | 1 | -0.43 | [-1.2, 0.29] |
| PHQ class 3 | PHQ class 2 | 77 | 17 | 0.04 | [-6.94, 7.01] | -0.01 | 20.1 | 0.99 | 1 | -0.003 | [-0.7, 0.5] |
| PHQ class 1 | PHQ class 2 | 10 | 17 | 5.16 | [-6.12, 16.45] | -0.96 | 18.1 | 0.35 | 1 | -0.39 | [-1.32, 0.42] |
| PHQ class 1 | PHQ class 3 | 10 | 77 | 5.13 | [-4.95, 15.1] | -1.14 | 10.2 | 0.28 | 1 | -0.43 | [-1.13, 0.39] |

**Table S6.** Differences in number of logins between different timepoints

| group 1 | group 2 | N1 | N2 | mean difference | CI | statistic | df | p | p adjusted | Cohen's D | CI |
| --- | --- | --- | --- | --- | --- | --- | --- | --- | --- | --- | --- |
| Logins wk 0-4 | Logins wk 4-8 | 221 | 221 | -2.86 | [-3.89, -1.84] | 7.66 | 220 | <0.001 | <0.001 | 0.515 | [0.38, 0.65] |
| Logins wk 0-4 | Logins wk 8-12 | 221 | 221 | -4.91 | [-5.86, -3.95] | 12 | 220 | <0.001 | <0.001 | 0.809 | [0.67, 0.94] |
| Logins wk 4-8 | Logins wk 8-12 | 221 | 221 | -2.05 | [-2.86, -1.23] | 6.84 | 220 | <0.001 | <0.001 | 0.46 | [0.32, 0.61] |

**Figure S1.** PHQ-9 classes (left) and GAD-7 classes (right)


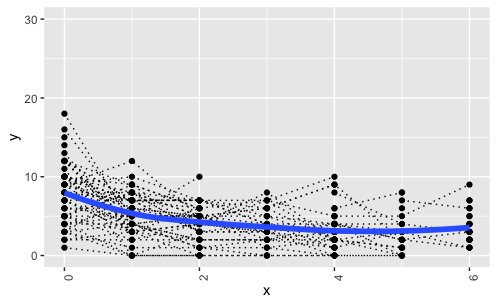

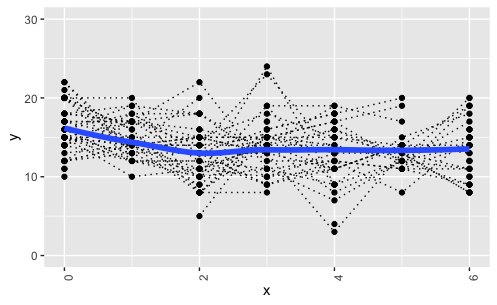

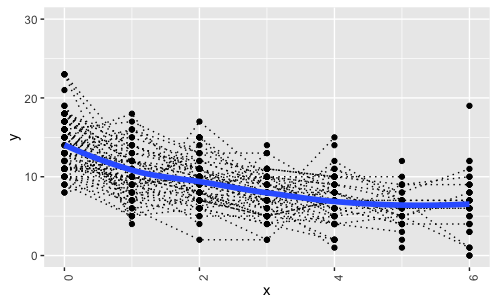

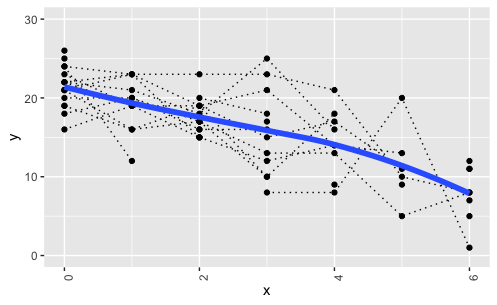

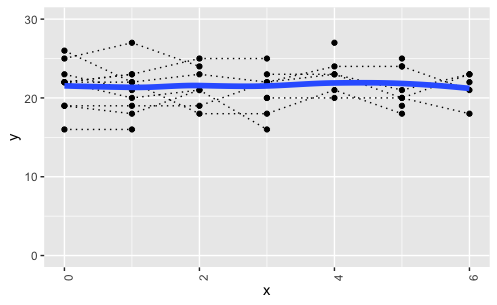


PHQ Class 1

PHQ Class 2

PHQ Class 3

PHQ Class 4

PHQ Class 5

GAD Class 1

GAD Class 2

GAD Class 3

GAD Class 4

GAD Class 5


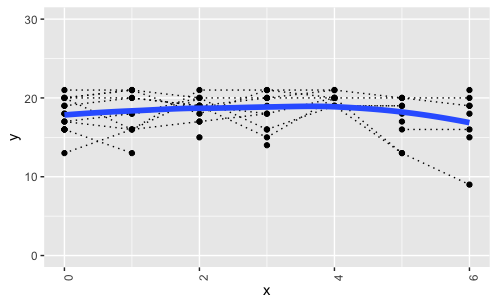

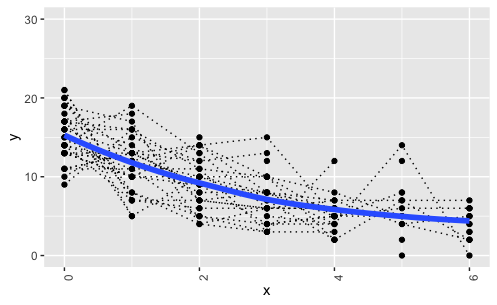

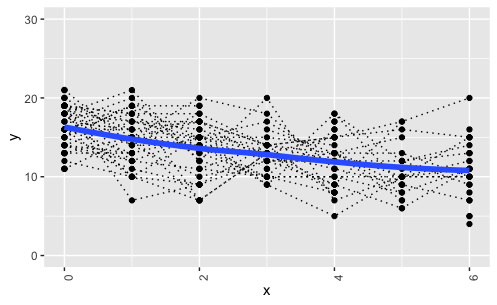

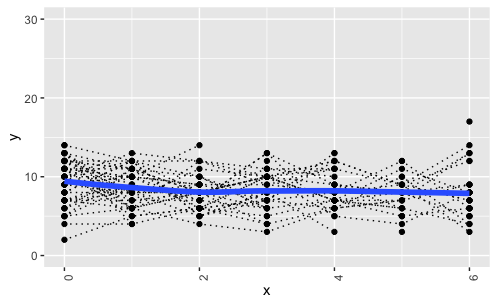

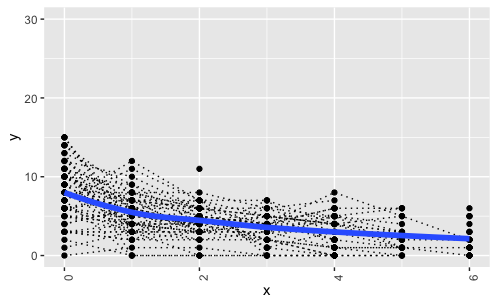


**Figure S2.** Example of SilverCloud programme homepage


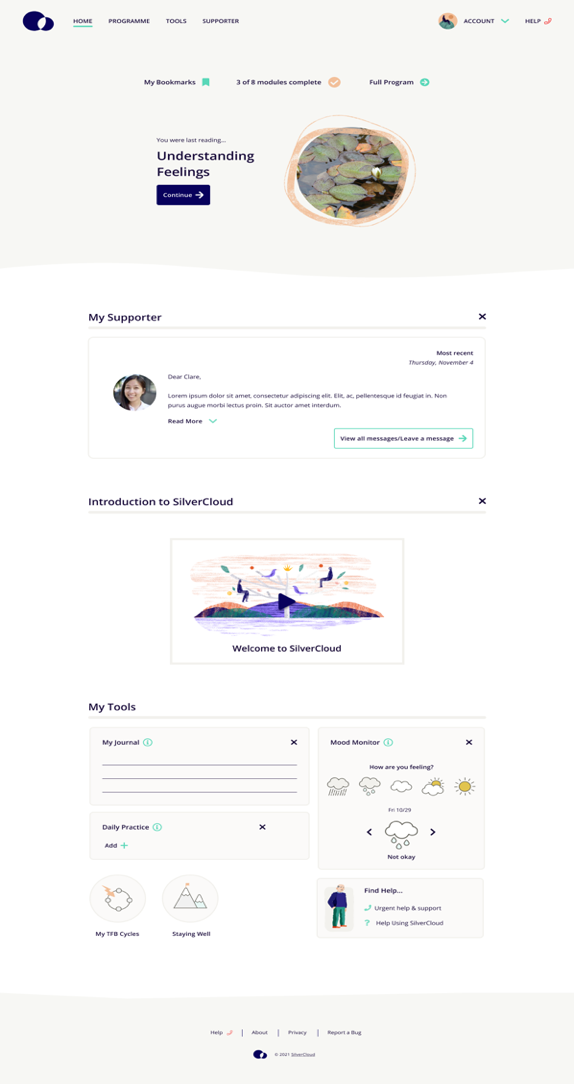


**Figure S3.** Example of SilverCloud programme tools page


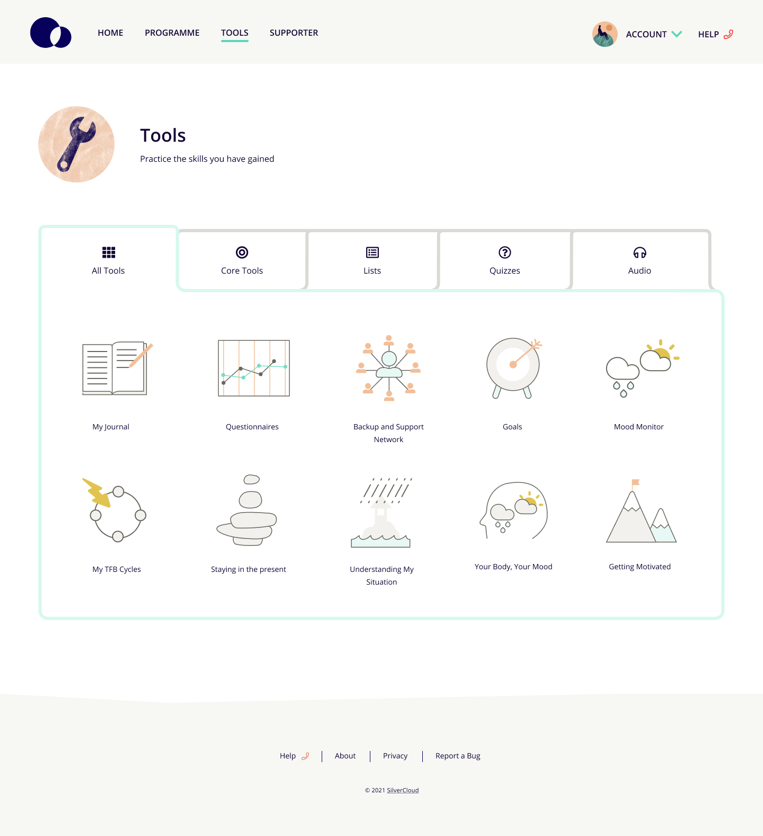

Supplement: Multimedia Appendix 1 [file mhealth_v11i1e41815_app1.docx]
